# Novel Insights into the Link Between Myeloperoxidase Modified LDL, LOX-1, and Neuroserpin in Stroke

**DOI:** 10.31083/j.rcm2412354

**Published:** 2023-12-15

**Authors:** Layal El-Hajjar, Elena Miranda, Marwan El-Sabban, Jalil Daher

**Affiliations:** ^1^Department of Anatomy, Cell Biology and Physiological Sciences, Faculty of Medicine, American University of Beirut, 1107 Beirut, Lebanon; ^2^Department of Biology and Biotechnologies ‘Charles Darwin’, Sapienza University of Rome, 00185 Rome, Italy; ^3^Department of Biology, Faculty of Arts and Sciences, University of Balamand, 100 El-Koura, Lebanon

**Keywords:** atherosclerosis, stroke, Mox-LDL, neuroserpin, endothelial dysfunction, lox-1

## Abstract

**Background:**

Cardiovascular disease that is caused by atherosclerosis is the leading cause of death worldwide. Atherosclerosis is primarily triggered by endothelial dysfunction and the accumulation of modified low-density lipoprotein (LDL) particles in the subendothelial space of blood vessels. Early reports have associated oxidized LDL with altered fibrinolysis and atherogenesis. It has been suggested that myeloperoxidase oxidized LDL (Mox-LDL) is involved in atherosclerosis because of its significant pathophysiological role in the modification of LDL *in vivo*. It has been equally demonstrated that Mox-LDL binds to the lectin-like oxidized low-density lipoprotein receptor-1 (lox-1) scavenger receptor which leads to the upregulation of inflammatory mediators in endothelial cells and the progression of cardiovascular disease. It has been also shown that neuroserpin, a member of the serine proteinase inhibitor (serpin) superfamily, has an important role at the level of fibrinolysis in the nervous tissue.

**Methods:**

Since little is known about the effects of Mox-LDL on endothelial cell fibrinolytic activity and the involvement of lox-1 in this process, our study aimed at evaluating the *in vitro *effects of Mox-LDL on neuroserpin release from human aortic endothelial cells (HAECs) and the role of lox-1 scavenger receptor in this context by relying on *lox-1* gene silencing in HAECs, culturing the cells in the presence of Mox-LDL, measuring their neuroserpin expression and release by quantitative polymerase chain reaction (qPCR) and enzyme-linked immunosorbent assay (ELISA), respectively, and assessing their fibrinolytic activity using the Euglobulin Clot Lysis Time (ECLT) method.

**Results:**

Our data show that Mox-LDL decreases endothelial cell fibrinolytic capacity by upregulating neuroserpin in *lox-1* knockdown cells.

**Conclusions:**

Lox-1 protects the endothelial cells from a Mox-LDL-induced decrease in pro-fibrinolytic capacity, which has important consequences in the context of stroke.

## 1. Introduction

Cardiovascular diseases (CVDs) include an extensive array of diseases that affect the function and structure of blood and heart tissues, and can be grouped into cerebrovascular disease (CD), coronary artery disease (CAD), and peripheral artery disease (PAD) [1]. CVDs are caused by atherosclerosis which is a progressive chronic inflammatory condition characterized by accumulation of oxidized forms of low-density lipoprotein (LDL) in the subendothelial space inside the intimal layer of arteries [2]. Although the mechanisms by which LDL is oxidized *in vivo* remain controversial, LDL that is modified by myeloperoxidase (MPO) is suggested as the most pathophysiological model of LDL oxidation [3, 4].

Meanwhile, it has been proposed that dysregulation in endothelial cell fibrinolytic capacity may negatively affect the course of atherosclerosis and that myeloperoxidase oxidized LDL (Mox-LDL) may have a role in altering endothelial cell pro-fibrinolytic activity [5, 6]. Endothelial cells feed the process of fibrinolysis by secreting multiple plasminogen activators and inhibitors and by expressing specific receptors that bind to those factors modulating their activity. Consequently, any interference with endothelial gene expression at this level can lead to endothelial dysfunction (ED) and has possible implications in the context of CAD and CD [7].

ED has been linked to plaque rupture and stroke and, as far as Mox-LDL is concerned, we have previously reported that Mox-LDL activates ED by binding to the lectin-like oxidized low-density lipoprotein receptor-1 (lox-1) scavenger receptor [8]. Lox-1 is a type II membrane protein receptor with a typical C-lectin structure at the C-terminus [9]. The binding of Mox-LDL to lox-1 leads to an increase in its expression and the induction of inflammatory pathways enhancing ED [8].

Concerning fibrinolysis, endothelial cells secrete tissue plasminogen activator (tPA) which is the predominant plasminogen activator in the blood that is responsible for converting plasminogen into plasmin and mediating thrombolysis; likewise, tPA has been used as the most common agent for thrombolysis treatment of patients with acute ischemic stroke. Neuroserpin, a member of the serine proteinase inhibitor (serpin) superfamily and a major inhibitor of tPA, plays a critical role in shaping the fibrinolytic response in the nervous system because of its function as a modulator of tPA activity [10, 11, 12]. In the context of stroke, tPA has a dual activity: while its thrombolytic function in intravascular settings is beneficial, its extravascular effects on ischemic neurons are very harmful, promoting multiple events that are associated with cell death and synaptic plasticity [10, 13]. In agreement with this, it has been shown that neuroserpin exerts neuroprotective properties in pathologies such as cerebral ischemia by preventing the excessive activity and adverse effects of tPA on parenchymal tissue [14].

Although research has mainly focused on the role of lox-1 in CAD, data on its involvement in ischemic stroke are gradually accumulating in recent clinical and epidemiological studies [15]. Interestingly, it has been recently shown that the incidence of ischemic stroke increases with higher circulating levels of lox-1 which could be considered a risk marker for developing CD [16]. It has been equally demonstrated that acetylsalicylic acid, a conventional medication used to prevent stroke, has an important role in inhibiting lox-1-mediated oxidized LDL signaling pathways in human endothelial cells [17]. Moreover, multiple studies suggest that the overexpression of *lox-1* in endothelial cells increases stroke size *in vivo* and that its deletion has a protective effect on stroke and spontaneous brain damage in stroke-prone hypertensive rats [17, 18].

Therefore, the goal of this study was to investigate the relationship between Mox-LDL and neuroserpin in the context of stroke and the role of lox-1 in this context.

## 2. Materials and Methods

### 2.1 Cell Culture

EBM-2 Basal Medium (supplemented with EGM-2 SingleQuots™ Kit supplements and growth factors) (Lonza, Basel, Switzerland) was used to culture human aortic endothelial cells (HAECs; kindly provided by Pr. El-Sabban Lab, American University of Beirut, Lebanon) that were incubated at 37 °C in a humidified incubator (95% air, 5% ${\mathrm{CO}}_{2}$). Cells were tested for *mycoplasma* using the LookOut *mycoplasma* Detection Kit (MP0035; Sigma-Aldrich, Saint Louis, MO, USA) following the manufacturer’s instructions.

### 2.2 Oxidation of LDL

As previously described, in order to oxidize LDL (L7914; Sigma-Aldrich, Saint Louis, MO, USA), 1.6 mg LDL (final concentration: 0.8 mg/mL in phosphate buffer saline (PBS), pH 7.4) was mixed with hydrochloric acid (HCl) (1 M, 8 µL), MPO (11.11 × ${\mathrm{10}}^{-6}$ M, 45 µL) and $H_{2}$$O_{2}$ (0.05 M, 40 µL). Then, PBS (pH 7.4) with 1 g/L of ethylenediaminetetraacetic acid (EDTA) was added to reach a final volume of 2 mL; LDL was filtered on a deslating column (17-0851-01; Cytiva, Freiburg, Germany) after MPO treatment. In this condition, the molar ratio of oxidant/lipoprotein was equal to 625:1 [19, 20].

### 2.3 Transfection

As per the manufacturer’s protocol, 5 nM non-targeting small interfering RNA (siRNA) (Silencer Select Negative Control, Ambion Applied Biosystems, Austin, TX, USA) or LOX-1 specific oxidized low density lipoprotein receptor 1-siRNA (OLR1-siRNA) (Silencer Select Validated siRNA ID s9843, Ambion Applied Biosystems, Austin, TX, USA) were used in order to transfect 20,000 ${\mathrm{cells/cm}}^{2}$ using Hiperfect Transfection Reagent (Qiagen, Hilden, Germany). Briefly, on the day of transfection, cells were incubated under normal conditions (37 °C and 5% ${\mathrm{CO}}_{2}$) after being seeded in 24-well plates in 100 µL complete medium (with fetal bovine serum and antibiotic). Then, 3 µL of transfection reagent were mixed with 37.5 ng of siRNA that were diluted in 100 µL of serum-free medium. To allow the formation of transfection complexes, samples were incubated for 5–10 min at room temperature. In order to ensure uniform distribution of the transfection complexes, complexes were added drop-wise into the cells and mixed gently. After incubation under normal growth conditions, cells were monitored for gene silencing after 24 h of transfection before proceeding with subsequent treatments.

### 2.4 Mox-LDL Treatment

Two 6-well plates were used in order to seed normal and *lox-1*-knockdown HAECs at 30,000 ${\mathrm{cell/cm}}^{2}$. Prior to enzyme-linked immunosorbent assay (ELISA), one row of each plate was either treated with Mox-LDL (100 µg/mL) or left untreated.

### 2.5 RNA Extraction and Quantitative Polymerase Chain Reaction (PCR)

Nucleospin® RNA II Kit (Machery-Nagel, Duren, Germany) was used to isolate total RNA from cells and a Nanodrop was used to measure total RNA concentrations and A260/A280. For reverse transcription, 1 µg of total RNA was converted into complementary DNA (cDNA) using iScript™ cDNA Synthesis Kit (ThermoFisher, Vilnius, Lithuania). Then, iQ SYBR Green Supermix was utilized in order to proceed with Quantitative PCR (qPCR) in a CFX96 system (Bio-Rad Laboratories, Hercules, CA, USA) using the sets of primers listed in Table 1. The standard cycling conditions were as follows: 95 °C for 5 min, followed by 40 cycles of 95 °C for 10 s, 57 °C for 30 s and 72 °C for 30 s. SDS 2.3 relative quantification manager software was used in order to analyze the results. Glyceraldehyde-3-phosphate dehydrogenase (*GAPDH*) was used as a reference gene and the comparative threshold cycle values were normalized against it. To ensure quantitative accuracy, qPCR was performed in triplicate. In order to calculate the relative fold change in gene expression after normalization, the $2^{-\Delta \,\Delta \,\text{Cq}}$ method was applied.

**Table 1.** **List of primers**.

Gene	Primer sequence
*SERPINI1* (*neuroserpin*)	F: 5′-AGGATGGCTGTGCTGTATCC-3′
	R: 5′-GTTTCAGGATGCATGACTCG-3′
*GAPDH (glyceraldehyde-3-phosphate dehydrogenase*)	F: 5′-TGGTGCTCAGTGTAGCCCAG-3′
	R: 5′-GGACCTGACCTGCCGTCTAG-3′

### 2.6 Enzyme-Linked Immunosorbent Assay (ELISA)

For cytokine analysis, the supernatants from HAEC cultures, that were transfected with *lox-1* siRNA or negative control siRNA and that were either treated with Mox-LDL or left untreated, were collected and stored at –80 °C. A commercially available sandwich ELISA kit (MBS9502108; MyBioSource, San Diego, CA, USA) was used in order to assess neuroserpin levels in the HAEC culture supernatants. The samples were processed in duplicates and measured at 450 nm on a micro-plate reader (Biotek, Winooski, VT, USA) as per the manufacturer’s instructions.

### 2.7 Assessment of Fibrinolysis 

Following a method previously described [21], 30,000 HAECs were grown on a polyethelene terephthalate (PET) microporous membranes that were coated with Type I collagen for 12 h in glass micro-cuvettes and incubated to reach confluence. Then, the cells were incubated for 24 h in the presence or absence of Mox-LDL at 100 µg/mL. Afterwards, the medium was discarded and the cells were washed with Hank’s Balanced Salt Solution (HBSS) three times before proceeding with the fibrinolytic test.

3.6 mL of deionized water and 300 µL of acetic acid (0.25%) were added to 400 µL of plasma (P9523; Sigma-Aldrich, Saint Louis, MO, USA) at a final pH of around 5. The samples were put into melting ice for 20 min and then centrifuged at 4000 g for 10 min at 4 °C. After discarding the supernatant, the remaining pellet was re-suspended in 400 µL of HBSS (HEPES 25 Mm, pH 7.3).

250 µL of euglobulin fraction were injected onto the cell-seeded glass micro-cuvettes that were placed in the spectrophotometer. In order to initiate clot formation, 50 µL of thrombin (T6884; Sigma-Aldrich, Saint Louis, MO, USA) was added to the micro-cuvettes, and the Euglobulin Clot Lysis Time (ECLT) was recorded and expressed in minutes.

### 2.8 Statistical Analysis

GraphPad Prism software (version 6.0; GraphPad Software, Inc., San Diego, CA, USA) was used in order to perform statistical analysis. Data were expressed as the mean ± standard error mean (SEM) and *p* < 0.05 was considered to show a statistically significant difference. Student’s *t*-test and one-way analysis of variance test (ANOVA) followed by Tukey’s multiple comparison post hoc test were used in order to study statistical significance.

## 3. Results

### 3.1 The Effect of Mox-LDL and LOX-1 Knockdown on Neuroserpin Secretion

**Fig. 1.**
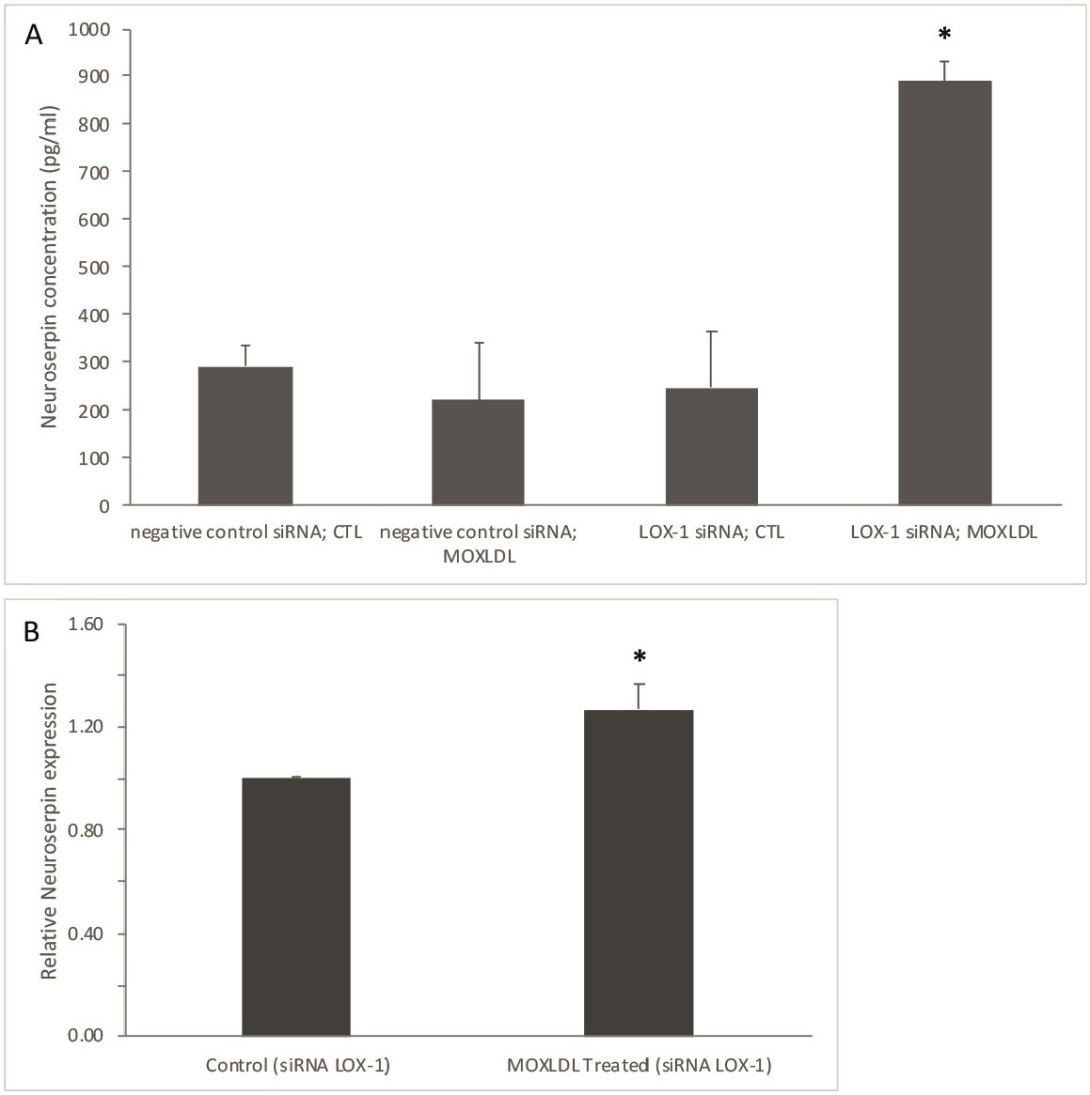
**Measurement of neuroserpin release and expression in Mox-LDL treated LOX-1 silenced HAECs**. (A) ELISA was used in order to measure neuroserpin concentration in the culture supernatants of cells that were treated with mock medium (CTL) or Mox-LDL (MOXLDL) for 24 h after transfection with either negative control or LOX-1 siRNA. Bar graphs represent the mean values of three independent experiments. Error bars represent SEM. One-way ANOVA followed by Tukey’s multiple comparison post hoc test was used to calculate statistical significance. *p < 0.05. (B) Bar graphs representing neuroserpin mRNA expression in HAECs that were treated with mock medium (Control) or Mox-LDL (MOXLDL) for 24 h after transfection with LOX-1 siRNA, as detected by qPCR and normalized to GAPDH from 3 independent experiments. Data are presented as the mean ± SEM (n = 3) fold change in mRNA expression. Statistically significant differences were determined by Student’s *t*-test (*p < 0.05). Mox-LDL, myeloperoxidase oxidized LDL; LOX-1, lectin-like oxidized low-density lipoprotein receptor-1; HAECs, human aortic endothelial cells; ELISA, enzyme-linked immunosorbent assay; SEM, mean ± standard error mean; qPCR, Quantitative PCR; siRNA, small interfering RNA; LDL, low density lipoprotein; ANOVA, analysis of variance test; PCR, polymerase chain reaction; GAPDH, glyceraldehyde-3-phosphate dehydrogenase.

In order to determine the role that Mox-LDL and lox-1 may play in the context of stroke, we characterized the secretion profile of neuroserpin in HAECs that were either transfected with *lox-1* siRNA or negative control siRNA and then treated with Mox-LDL (MOXLDL) or left untreated (CTL). After validation of *lox-1 *silencing (**Supplementary Fig. 1**) in HAECs, analysis of the secretion profiles clearly showed that Mox-LDL treated *lox-1* knockdown cells presented significantly increased secretion levels of neuroserpin compared to untreated cells, and this effect was only seen upon *lox-1* knockdown (Fig. 1A). We confirmed this observation by quantitative PCR at the mRNA level and found that neuroserpin expression increased significantly after treating the *lox-1* silenced cells with physiological concentrations of Mox-LDL (Fig. 1B). Taken together, these results validate the role of Mox-LDL and lox-1 with regards to the upregulation of neuroserpin both at the mRNA and protein levels.

### 3.2 The Effect of Mox-LDL and LOX-1 on Fibrinolysis

**Fig. 2.**
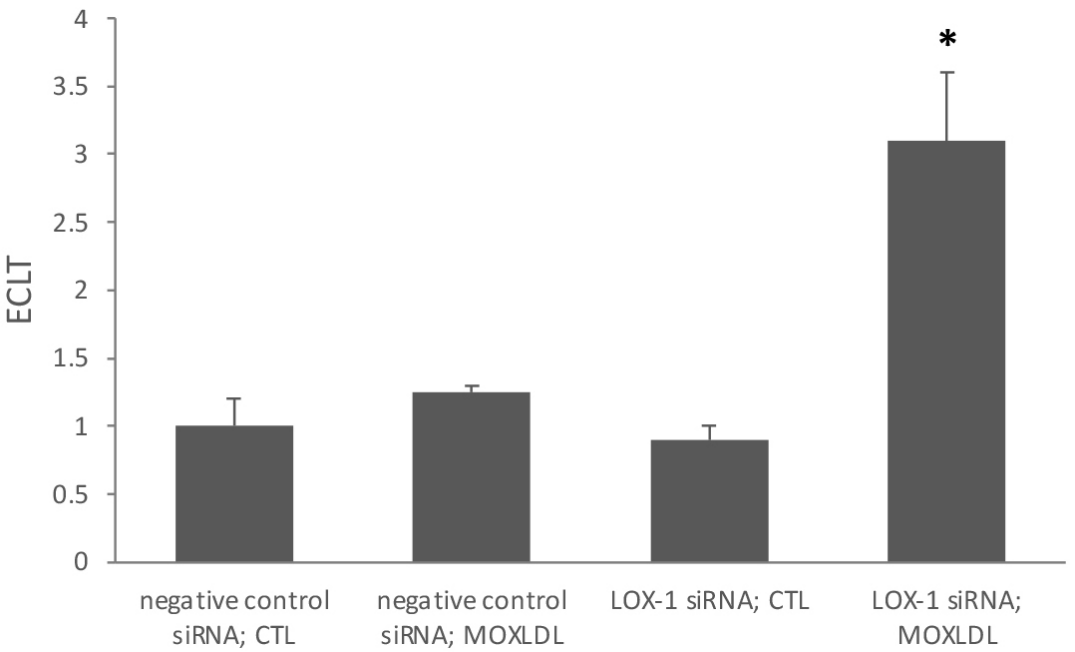
**Mox-LDL significantly alters the pro-fibrinolytic capacity of LOX-1-silenced HAECs**. The ECLT was measured in the presence of HAECs that were either transfected with negative control siRNA or LOX-1 siRNA and treated with mock medium or with Mox-LDL (100 µg/mL). Results are expressed as fold over control ratio. Bar graphs represent the mean values of three independent experiments. Error bars represent SEM. One-way ANOVA followed by Tukey’s multiple comparison post hoc test was used to calculate statistical significance. *p < 0.05. ECLT, Euglobulin Clot Lysis Time; SEM, mean ± standard error mean; siRNA, small interfering RNA; CTL, control non-treated; Mox-LDL, myeloperoxidase oxidized low density lipoprotein; LOX-1, lectin-like oxidized low-density lipoprotein receptor 1; HAECs, human aortic endothelial cells; ANOVA, analysis of variance test; MOXLDL, myeloperoxidase oxidized low density lipoprotein treated.

Next, we monitored the effect of Mox-LDL exposure and *lox-1* silencing on the process of fibrinolysis in endothelial cells by measuring the clot lysis time. Treatment with physiological concentrations of Mox-LDL (100 µg/mL) of *lox-1* knockdown HAECs led to a significant increase (more than 3 fold) in the lysis time as compared to other treatment conditions. The data show that endothelial cells are particularly susceptible to the anti-fibrinolytic effect of Mox-LDL when *lox-1* is not expressed (Fig. 2).

## 4. Discussion

In this study, the relationship between Mox-LDL, lox-1 and neuroserpin was investigated for the first time. Our data indicate that there is a potential link whereby the lox-1 scavenger receptor is possibly involved in affecting the fibrinolytic response of HAECs to physiological levels of Mox-LDL. Our results must be discussed by taking into consideration what has been reported concerning the role of Mox-LDL in atherosclerosis. The MPO model of LDL oxidation is pathophysiologically relevant to what happens *in vivo *and it has been confirmed by many immunohistochemical analyses that the MPO enzyme and its oxidized LDL products are present within the atherosclerotic lesions of patients with the disease [4, 22].

Mox-LDL was shown to have pro-inflammatory properties in different types of cells including endothelial cells where it triggers interleukin-8 (IL-8) release. The latter is instrumental during atherogenesis since it plays a crucial role in the recruitment of immune cells and smooth muscle cells to the growing plaque, enhancing inflammation and arterial remodeling [23, 24]. In the context of atherogenesis, macrophages accumulate Mox-LDL leading to the formation of foam cells which are considered as one of the hallmarks of the disease. Furthermore, Mox-LDL has been reported to activate monocytes and macrophages and enhance inflammation by increasing tumor necrosis factor-alpha (TNF-α) release [25]. Also, many clinical studies have linked myeloperoxidase and its modified LDL product to multiple atherosclerosis-related diseases including erectile dysfunction and kidney failure [26, 27, 28, 29].

On the other hand, lox-1, a lectin-like scavenger receptor, is expressed in endothelial cells and has been suggested as a receptor for multiple types of oxidized LDL particles including LDL that is modified by MPO. It has been reported that this scavenger receptor is highly expressed in atherosclerotic arteries of humans and is induced by many pro-atherogenic stimuli including dyslipidemia, angiotensin II, shear stress and diabetes [9, 30]. It was also proved that the lox-1 receptor is tightly linked to inflammatory processes that drive ED and enhance the development of atherosclerosis [31]. In our model, we have previously reported that Mox-LDL upregulates the expression of the lox-1 receptor which induces inflammatory signaling pathways leading to ED. We reported that Mox-LDL causes a dysfunction in the ability of endothelial cells to build vascular networks *in vitro* and that this effect was more profound when *lox-1* is silenced [8].

Meanwhile, we have also investigated the role of Mox-LDL at the level of fibrinolysis in endothelial cells. Our observations suggested that Mox-LDL delays pericellular fibrinolysis which can lead to endothelial cell dysfunction by enhancing fibrin deposition on the cells and increasing their permeability, which is critical in the context of an atheroma plaque formation [6]. Nonetheless, these observations were never linked to any effect of Mox-LDL on the expression level of major fibrinolytic factors that are secreted by endothelial cells including plasminogen activator inhibitor-1 (PAI-1), tPA, urokinase plasminogen activator (uPA) and their respective receptors tissue plasminogen activator receptor (tPAR) and urokinase plasminogen activator receptor (uPAR) [5]. On a different note, we also reported the involvement of Mox-LDL in impairing wound healing and increasing permeability in endothelial cells by upregulating micro RNA-22 (miR-22) and heme oxygenase 1 which may have serious implications in the context of atherosclerosis and the progression of the disease. Once again, uncertainties remain in the signal transduction pathway leading to dysfunction at this level [32].

In the present study, our initial results show that Mox-LDL treatment significantly increases neuroserpin release from HAECs (~3-fold) that are silenced for *lox-1*. Thus, our data suggest that lox-1 may have an indirect negative effect on neuroserpin expression which confirms its pro-atherogenic properties and potentially detrimental effects in the context of CD and stroke. Neuroserpin has been shown to have a neuroprotective role in brain ischemic regions. It has been reported that it colocalizes and reacts preferentially with its tPA target in the brain tissue. Interestingly, both *neuroserpin* and *tPA* expression are increased in cerebral ischemia where additional treatment with neuroserpin or its overexpression can result in a significant decrease in the size of ischemic areas [33, 34]. Moreover, it was also reported that neuroserpin levels are significantly higher in patients with Alzheimer’s disease, and immunohistochemical analysis has shown that tPA-neuroserpin complexes are seen in brain tissue from Alzheimer’s disease patients and are associated with amyloid plaques. In the brain, the inhibitory activity of neuroserpin and the reduced generation of plasmin may be responsible for decreasing the clearance of amyloid-beta, while the decline in tPA activity may also be directly linked to the impairment in synaptic activity and cognitive function [35, 36]. Outside the brain, it was also reported that neuroserpin is expressed in multiple types of organs including the heart, pancreas, skin, kidney and testis where it is responsible for selectively inhibiting the activity of tPA [37]. Again, in the context of cerebral ischemia, it was shown that tPA has a dual role; its beneficial effects might occur in few hours after the onset of ischemia and might have to do with its intravascular thrombolytic properties that reduce the extent of neurologic damage. Conversely, several studies have shown that tPA also exhibits a detrimental role in the nervous tissue where its extravascular and excessive effects have been linked to neuronal toxicity and death [13, 38, 39]. In the nervous system, the balance between neuroserpin and tPA is crucial and is important in the regulation of many processes including neuronal plasticity and death [40]. After a stroke, neuroserpin is endowed with neuroprotective effects seemingly because it widens the therapeutic window of tPA-beneficial thrombolytic activity and blocks its deleterious extravascular effects which can lead eventually to an overall decrease in stroke volume in a rat model of embolic stroke [10]. In humans, it has been equally reported that neuroserpin is neuroprotective in patients with ischemic stroke where higher neuroserpin levels were associated with better functional outcomes through lowering the secretion of inflammatory markers. This was also linked to a decrease in infarct size which could provide additional evidence of the protective effects of neuroserpin outcomes in cerebral ischemic patients. In this context, a decrease in serum levels of neuroserpin may be considered a predictive marker for clinical outcomes of stroke [12].

Recently, the lox-1 scavenger receptor has attracted much attention in research and studies of stroke and CD where current data support its involvement in cerebral ischemia through the disruption of the blood-brain barrier in a stroke-prone model of hypertensive rats [18]. Additional studies have highlighted the deleterious role of endothelial lox-1 in cerebral injury, where its increased expression is the precipitating cause of ischemic stroke in a middle cerebral occlusion mouse model of brain injury. In the latter model, overexpression of *lox-1* to unphysiological levels was associated with an increase in endothelial dysfunction and stroke size *in vivo * [17, 41].

Interestingly, our data indicate that *lox-1* knockdown cells are more susceptible to Mox-LDL-induced neuroserpin release which also correlates with a significant increase in the ECLT and thus, a decrease in the pro-fibrinolytic activity of HAECs. The latter observation is reminiscent of what was already reported regarding the negative effect of Mox-LDL at the level of fibrinolysis in endothelial cells [5, 6]. Overall, these results could eventually offer a hint to complete the paradigm of the anti-fibrinolytic effect of Mox-LDL where the latter enhances fibrin generation by binding to a still unknown receptor which will lead to an increase in neuroserpin secretion from endothelial cells and further inhibition of its tPA target. Although the exact pathway behind this phenomenon remains unclear, especially since Mox-LDL does not bind to LOX-1 to mediate its specific anti-fibrinolytic effects, the latter observation may be related to various mechanisms including the uptake of Mox-LDL by two different receptors. In this context, the knockdown of *lox-1* may be responsible for enhancing the binding of Mox-LDL to a different competitive receptor and, thus, the increase in the activation of a putative signaling pathway that involves neuroserpin release. Therefore, confirming the expression pattern of other potential receptors for Mox-LDL and dissecting the related signaling pathways is essential in order to better understand the effects of Mox-LDL in ED, more specifically in relation to the process of fibrinolysis. It is worth mentioning here that it has been reported that Lipoprotein a, an LDL-like particle with a structure that is similar to tPA, competes with plasminogen for its binding site which could lead to a reduction in fibrinolysis in endothelial cells and an increase in the risk of CVD [42]. Accordingly, Mox-LDL may also be involved in decreasing pericellular fibrinolysis in endothelial cells by competitively binding to the tPA receptor mediating its anti-fibrinolytic effects, although this hypothesis requires further evaluation.

**Fig. 3.**
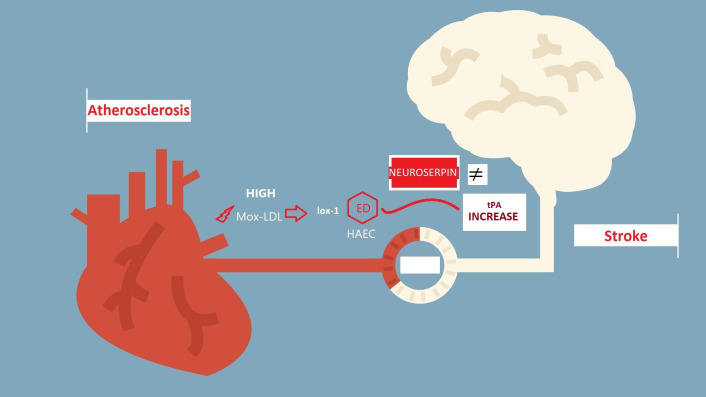
**In atherosclerosis, high Mox-LDL levels alter the process of fibrinolysis in HAECs by indirectly affecting neuroserpin release through binding to the scavenger receptor lox-1 which leads to endothelial cell dysfunction **. Lox-1 apparently plays a role in antagonizing Mox-LDL-induced decrease in pro-fibrinolytic activity in HAECs. These observations could shed more light onto the dual role that Mox-LDL may play during atherogenesis and stroke where it could be involved in regulating neuroserpin release and consequently the divergent effects of tPA on ischemic neurons by preferably binding to the lox-1 scavenger receptor. Mox-LDL, myeloperoxidase oxidized low density lipoprotein; lox-1, lectin-like oxidized low-density lipoprotein receptor 1; HAECs, human aortic endothelial cells; ED, endothelial dysfunction; tPA, tissue plasminogen activator.

In summary, Mox-LDL affects the process of fibrinolysis in HAECs by indirectly regulating neuroserpin release through binding the scavenger receptor lox-1 where the latter apparently protects the endothelial cells from Mox-LDL induced decrease in pro-fibrinolytic capacity (Fig. 3). These observations could have important implications regarding the negative and dual role that Mox-LDL may play in atherogenesis and CD where it may be involved in regulating the divergent effects of tPA and decreasing the inhibitory and neuroprotective capacity of neuroserpin through preferably binding to the lox-1 scavenger receptor; both processes are deleterious to ischemic neurons after stroke. Considering the instrumental role that the Mox-LDL-LOX-1 signaling axis may have in the development and advancement of atherosclerosis and other related diseases, mainly CD, targeting this interaction would be extremely promising for the development of anti-atherogenic therapeutic strategies to prevent and manage ischemic strokes and their complications. Those strategies could involve targeting *lox-1* for gene silencing by relying on RNA interference methods or blocking this scavenger receptor by specifically designed neutralizing monoclonal antibodies that bind to it. Furthermore, our results pave the way for future studies that would tackle a battery of scavenger receptors to better decipher Mox-LDL signaling pathways in ED and CVD. Finally, this line of research should attract much more attention from the scientific community given the fact that our model of LDL oxidation is highly relevant to what happens *in vivo* during disease initiation and progression as previously mentioned. Thus, in a clinical setting, there should be much more effort aimed at designing, developing and assessing drugs that could be successful in inhibiting the MPO enzyme and its Mox-LDL product and, thus, could be used as an alternative treatment for CVD and CD. On this account, several drugs were previously tested and identified as potential inhibitors of MPO including the antipsychotic drug fluoroalkylindole and the anti-inflammatory, antioxidant and antimicrobial drug benzoic acid hydrazide that were shown to have potent and promising effects in reversibly inhibiting MPO [43]. Also, lipid lowering drugs such as statins are being rediscovered lately as a treatment to better target oxidized LDL effects in atherosclerosis; for instance, it has been shown that the drug Simvastain may play an important role in atheroma plaque stabilization by regulating oxidized LDL induced autophagy in macrophages and reducing lipid loading inside atherosclerostic lesions [44].

## Data Availability

All data generated or analyzed during this study are included in this article.

## Supplementary Material

Supplementary material associated with this article can be found, in the online version, at https://doi.org/10.31083/j.rcm2412354.
