# Supplementary material for: Novel Insights into the Link Between Myeloperoxidase Modified LDL, LOX-1, and Neuroserpin in Stroke
Source: Rev Cardiovasc Med. 2023 Dec 15;24(12):354. doi: 10.31083/j.rcm2412354 (PMC11262435; doi:10.31083/j.rcm2412354)
Supplement: Supplementary file 1 [file 2153-8174-24-12-354-s1.doc]

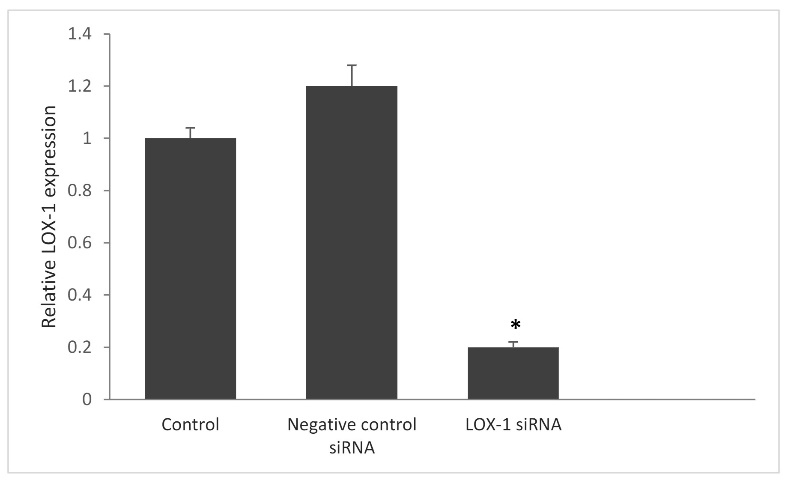


**Supplementary Fig. 1. The effect of gene silencing on the LOX-1 gene expression in HAECs.** Bar graph representing mRNA expression in HAECs in different treatment conditions that were normalized to the untreated (Control) condition. Data were generated by qPCR and GAPDH gene expression was used for normalization. Mean ±SEM fold change from 3 independent experiments performed in duplicate. ** P* ˂ 0.05 (ANOVA, Tukey’s multiple comparison test).
